# Epigenetic regulation by G9a/GLP complex ameliorates amyloid‐beta 1‐42 induced deficits in long‐term plasticity and synaptic tagging/capture in hippocampal pyramidal neurons

**DOI:** 10.1111/acel.12634

**Published:** 2017-06-30

**Authors:** Mahima Sharma, Tobias Dierkes, Sreedharan Sajikumar

**Affiliations:** ^1^ Department of Physiology Yong Loo Lin School of Medicine National University of Singapore Block MD9, 2 Medical Drive Singapore 117 597 Singapore; ^2^ Neurobiology/Aging Program Life Sciences Institute (LSI) National University of Singapore #04‐44, 28 Medical Drive Singapore 117 456 Singapore; ^3^ Institute of Innate Immunity Biomedical Centre University hospital Bonn Sigmund‐Freud‐Str. 25 Bonn 53127 Germany; ^4^ Division of Cellular Neurobiology Zoological Institute Technical University Braunschweig Braunschweig Germany

**Keywords:** amyloid β oligomer, BDNF, epigenetics, histone lysine‐methyltransferase, long‐term potentiation, synaptic tagging/capture

## Abstract

Altered epigenetic mechanisms are implicated in the cognitive decline associated with neurodegenerative diseases such as in Alzheimer's disease (AD). AD is the most prevalent form of dementia worldwide; amyloid plaques and neurofibrillary tangles are the histopathological hallmarks of AD. We have recently reported that the inhibition of G9a/GLP complex promotes long‐term potentiation (LTP) and its associative mechanisms such as synaptic tagging and capture (STC). However, the role of this complex in plasticity impairments remains elusive. Here, we investigated the involvement of G9a/GLP complex in alleviating the effects of soluble Amyloid‐β 1‐42 oligomers (oAβ) on neuronal plasticity and associativity in the CA1 region of acute hippocampal slices from 5‐ to 7‐week‐old male Wistar rats. Our findings demonstrate that the regulation of G9a/GLP complex by inhibiting its catalytic activity reverses the amyloid‐β oligomer‐induced deficits in late‐LTP and STC. This is achieved by releasing the transcription repression of the brain‐derived neurotrophic factor (*Bdnf*) gene. The catalytic inhibition of G9a/GLP complex leads to the upregulation of *Bdnf* expression in the slices treated with oAβ. This further ensures the availability of BDNF that subsequently binds its receptor tyrosine kinase B (TrkB) and maintains the late‐LTP. Furthermore, the capture of BDNF by weakly activated synapses re‐establishes STC. Our findings regarding the reinstatement of functional plasticity and associativity in AD‐like conditions provide the first evidence for the role of G9a/GLP complex in AD. We propose G9a/GLP complex as the possible target for preventing oAβ‐induced plasticity deficits in hippocampal neurons.

## Introduction

Epigenetic regulation plays a critical role in the process of learning and memory (Day & Sweatt, 2011; Jarome & Lubin, 2014), and dysregulation of these mechanisms underlies cognitive decline associated with neurodegenerative diseases such as Alzheimer's disease (AD) (Cacabelos & Torrellas, 2015; Maloney & Lahiri, 2016). Various epigenetic alterations have been identified in AD (Cadena‐del‐Castillo *et al*., 2014; Cacabelos & Torrellas, 2015; Grinan‐Ferre *et al*., 2016; Klein *et al*., 2016). Higher levels of DNA methylation and DNA hydroxymethylation are reported in different mouse models of AD (Cadena‐del‐Castillo *et al*., 2014; Cong *et al*., 2014). HDAC inhibitors exert a protective effect in AD (Klein *et al*., 2015; Krishna *et al*., 2016), indirectly suggesting the importance of regulation of histone acetylation during the cognitive decline. Histone methylation is thought to play an important role in AD as well. The G9a/GLP complex, along with other enzymes, regulating this histone modification is widely implicated in learning and memory processes (Schaefer *et al*., 2009; Maze *et al*., 2010; Fischer, 2014). The G9a/GLP complex is a histone lysine‐methyltransferase complex that predominantly dimethylates lysine 9 residue of Histone 3 (H3K9me2) (Tachibana *et al*., 2005). Catalytic inhibition of this epigenetic complex was recently reported to promote long‐term potentiation (LTP), a cellular correlate of memory and its associative mechanisms such as synaptic tagging and capture (STC) (Sharma *et al*., 2016). However, the role of this complex in plasticity impairment still remains elusive.

AD is a progressive neurodegenerative disorder, whose pathological hallmarks are β‐amyloid plaques and Tau/neurofibrillary tangles, and is characterized by irreversible memory loss. Amyloid‐β 1–42 (Aβ) is responsible for the synaptic failure (Chen *et al*., 2000; Sheng *et al*., 2012; Selkoe & Hardy, 2016) that is associated with cognitive decline and forms the basis of clinical AD phenotype (Sheng *et al*., 2012). Exogenous application of Aβ 1–42 impairs synaptic plasticity *in vitro* as well as *in vivo*, which underlies the process of memory formation (Ma *et al*., 2014; Lei *et al*., 2016). Deficits in associative learning have also been reported in the preclinical form of AD (Jiang *et al*., 2015; Quenon *et al*., 2015). STC model explains the formation of memories in an associative and time‐dependent manner (Frey & Morris, 1997; Redondo & Morris, 2011). This model proposes that a ‘tag’ set by a weak stimulus or a weak memory trace ‘captures’ the plasticity factors induced by a strong stimulus or a strong memory trace in two independent synaptic inputs of the same neuronal population. This tag‐PRP interaction results in the consolidation of memory (Redondo & Morris, 2011).

Brain‐derived neurotrophic factor (BDNF) is considered as one of the major molecular mediators of functional and morphological synaptic plasticity (Pang & Lu, 2004; Rex *et al*., 2007). It is widely recognized plasticity factor that maintains late‐LTP, late‐LTD and STC (Korte *et al*., 1995; Sajikumar & Korte, 2011). Studies suggest that BDNF and/or expression of its receptors alter during aging and neurodegenerative diseases (Beeri & Sonnen, 2016; Buchman *et al*., 2016). Higher BDNF levels correlate with slower cognitive decline in aging and vice versa (Michalski *et al*., 2015; Buchman *et al*., 2016). BDNF is also reported to be neuroprotective against toxic effects of Aβ peptides (Arancibia *et al*., 2008; Caccamo *et al*., 2010).

In the present study, we investigated the role of G9a/GLP complex in alleviating the effects of soluble amyloid‐β oligomers 1–42 (oAβ) on late‐LTP and STC in the CA1 region of acute hippocampal slices from 5‐ to 7‐week‐old male Wistar rats. We found that the pharmacological inhibition of G9a/GLP complex activity prevents the amyloid‐β oligomer‐induced deficits in late‐LTP and STC. Our study provides the first evidence of the beneficial effects of inhibiting the G9a/GLP complex activity to restore plasticity and associativity in the CA1 region of hippocampal pyramidal neurons. We further report that the regulation of G9a/GLP complex by inhibiting its activity increases the BDNF signaling, which mediates the restoration of long‐term plasticity and associativity.

## Results

### Soluble Aβ (1–42) oligomer does not affect the short‐term plasticity, but significantly impairs the late‐LTP

**Figure 1:**
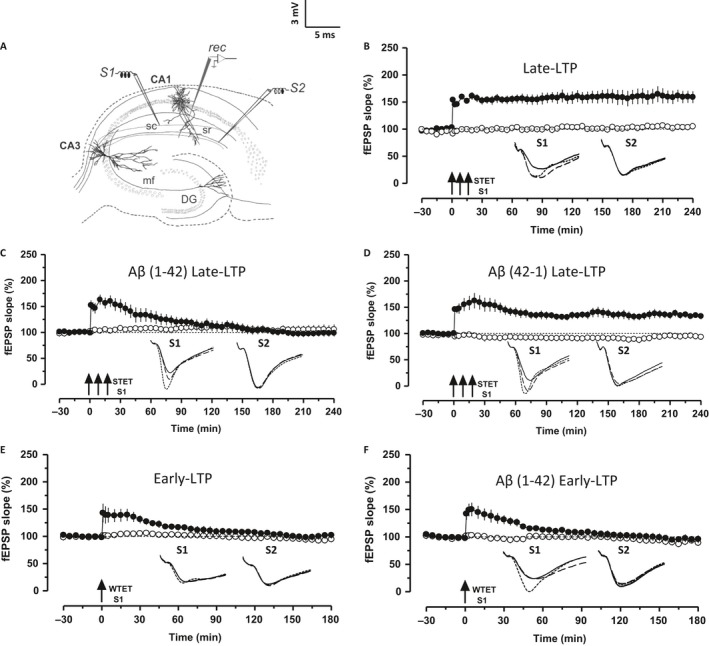
A*β* 1–42 impairs late‐LTP but not early‐LTP. (A) Schematic representation of the positioning of electrodes in the CA1 region of a transverse hippocampal slice. Recording electrode (rec) positioned in CA1 apical dendrites was flanked by two stimulating electrodes S1 and S2 in stratum radiatum (sr) to stimulate two independent Schaeffer collateral (sc) synaptic inputs to the same neuronal population. (B) Application of strong tetanization (STET) in S1 (filled circles) resulted in late‐LTP. The control potentials in S2 (open circles) were relatively stable (*n* = 6). (C) Hippocampal slices pretreated with A*β* 1–42 (Aβ, 200 nm) for 2 h during the incubation period failed to show late‐LTP after STET in S1 (filled circles) (*n* = 8). (D) A*β* 42–1 (200 nm)‐treated slices expressed late‐LTP after the application of STET (*n* = 9). (E–F) Induction of early‐LTP in S1 (filled circles) using a weak tetanization (WTET) protocol in both the control slice (*n* = 6) and A*β*‐treated slices (*n* = 7) resulted in early‐LTP. Control potentials from S2 remained stable during the recorded period (open circles) in all the cases. Analog traces represent typical field EPSPs of inputs S1 and S2 15 min before (solid line), 30 min after (dotted line) tetanization and at the end of the recording (dashed line). Solid arrow indicates the time point of STET or WTET of the corresponding synaptic input. All data are plotted as mean ± SEM. Error bars indicate SEM. Calibration bar for all analog sweeps: 3 mV per 5 ms.

We first confirmed that hippocampal slices treated with exogenous Aβ 1–42 impair late‐LTP in our experimental conditions. Aβ 1–42 oligomer (oAβ, 200 nm) was bath applied to the hippocampal slices for 2 h during the incubation period similar to that of earlier reports (Ronicke *et al*., 2011). After recording a stable baseline of 30 min, application of strong tetanization (STET) to the synaptic input S1 (Fig. 1C, filled circles) resulted in potentiation that waned over next 1–2 h, unlike the control late‐LTP which maintained for 4 h (Fig. 1B, filled circles; Wilcox test, *P = *0.028; *U*‐test *P = *0.005). In Fig. 1C, the potentiation in S1 was statistically significant up to 60 min (Wilcox test, *P = *0.048) or up to 40 min (*U*‐test, *P = *0.0135). Basal synaptic transmission was not affected by oAβ, as is evident by the baseline recordings in S2 (Fig. 1C, open circles). To rule out the nonspecific peptide effects of Aβ 1–42 on the impairment of late‐LTP, late‐LTP was induced in presence of Aβ 42–1 (200 nm), which maintained up to 4 h (Wilcox test, *P = *0.0076; *U*‐test, *P = *0.0008) in S1 (Fig. 1D, filled circles). The application of weak tetanization (WTET) in both the control hippocampal slices (Fig. 1E) and the oAβ‐treated slices (Fig. 1F) resulted in an early‐LTP. Potentiation in both E and F was statistically significant for 55–65 min (Fig. 1E, Wilcox test, *P* = 0.046, *U*‐test = 0.065; Fig. 1F, Wilcox test, *P = *0.03, *U*‐test, *P = *0.087). Together, these findings suggest that Aβ 1–42 abrogates late plasticity without affecting early plasticity.

### Regulation of G9a/GLP complex by inhibiting its activity rescues the Aβ 1–42 induced deficits in LTP

**Figure 2:**
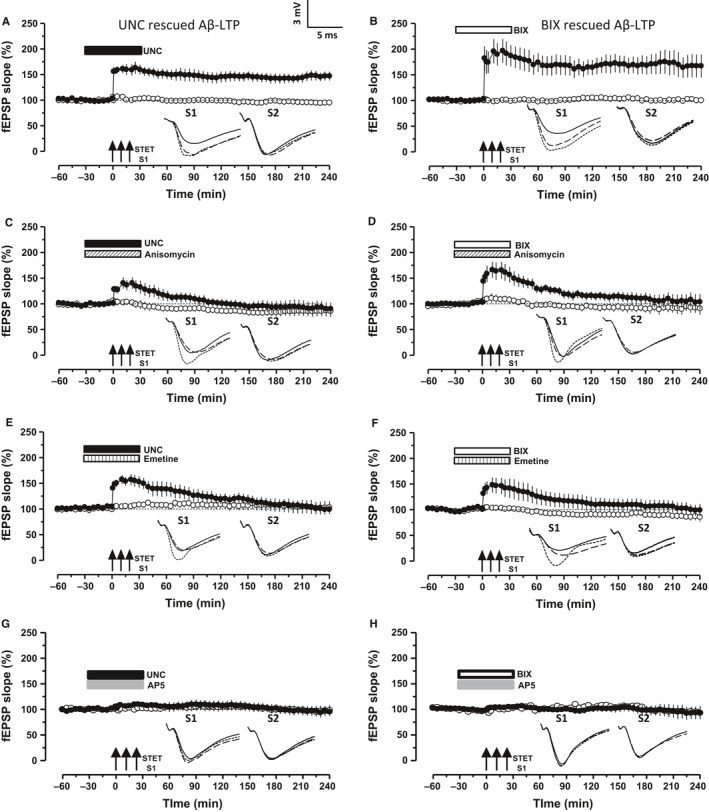
Inhibition of G9a/GLP complex activity rescues Aβ‐induced deficits in LTP. (A) Aβ‐treated hippocampal slices expressed late‐LTP in the presence of UNC 0638 (UNC, 150 nm), where drug was bath applied 30 min before and after the LTP induction by STET (filled circles, *n* = 7). (B) Same experiment as in A, but with another G9a/GLP inhibitor BIX 01294 (BIX, 500 nm,* n* = 6). The control potentials were stable throughout the recording (open circles). (C‐F) Co‐application of UNC or BIX with protein synthesis inhibitors anisomycin (25 μm) or emetine (20 μm) prevented the rescue of late‐LTP (filled circles) in the hippocampal slices pretreated with Aβ (C, *n* = 6, D, *n* = 6, E, *n* = 9 and F, *n* = 6). Control potentials were stable in all the cases (open circles). (G‐H) Effect of the NMDA receptor blocker AP‐5 on G9a/GLP inhibition mediated rescue of Aβ‐induced plasticity impairment: STET in the presence of AP‐5 (50 μm) along with either UNC (G, *n* = 6) or BIX (H, *n* = 5) prevented the establishment of late‐LTP (filled circles); the control recording of S2 remained stable during the recorded period (open circles). Error bars indicate ± SEM. Symbols and analog traces as in Fig. 1.

We have reported recently that the repression of G9a/GLP complex activity reinforces early‐LTP to late‐LTP in a protein synthesis‐dependent manner (Sharma *et al*., 2016). We were intrigued to know whether the modulation of the activity of this epigenetic complex could rescue the plasticity deficits induced by exogenous oAβ. To test this idea, the pharmacological inhibitor of G9a/GLP complex UNC0638 (UNC, 150 nm) or BIX 01294 (BIX, 500 nm) was bath applied to the oAβ‐treated slices for a duration of 1 h, spanning from 30 min pre–post late‐LTP induction by STET. Application of STET in S1 in both the conditions resulted in late‐LTP (Fig. 2A,B, filled circles). Statistically significant potentiation was observed in both the cases till the end of the recording period (Fig. 2A, Wilcox test, *P *=* *0.018, *U*‐test, *P = *0.002; Fig. 2B, Wilcox test, *P = *0.028, *U*‐test, *P = *0.008). The baseline potentials in S2 (open circles) were stable throughout the recording period.

Next, we investigated the requirement of protein synthesis and NMDA receptor activity for the rescued plasticity to confirm whether the rescue occurred in a physiological manner. Bath application of two structurally distinct protein synthesis inhibitor anisomycin (ANI, 25 μm) or emetine (EME, 20 μm) together with UNC (Fig. 2C,E) or BIX (Fig. 2D,F) during the induction of late‐LTP prevented its late maintenance (S1, filled circles, Fig. 2C–F; ANI, Fig. 2C,D; EME, Fig. 2E,F). Potentials in S1 of Fig. 2C stayed statistically significant up to 30 min after STET (Wilcox test, *P = *0.046) or up to 21 min (*U*‐test, *P = *0.03) and in Fig. 2D up to 110 min (Wilcox test, *P = *0.028, *U*‐test, *P = *0.045). The experimental series in which emetine was co‐applied with UNC showed statistically significant potentiation lasting up to 70 min (Fig. 2E, Wilcox test, *P = *0.04) or up to 60 min (*U*‐test, *P = *0.04), whereas in Fig. 2F the significant potentiation lasted up to 55 min (Fig. 2F, Wilcox test, *P = *0.046) or up to 30 min (*U*‐test, *P = *0.03). The control input S2 remained stable throughout the recording period (Fig. 2C–F, open circles). NMDA receptor dependency was tested using its antagonist AP‐5 (50 μM). AP‐5 was co‐applied with either UNC or BIX for 60 min (Fig. 2G,H). STET 30 min after the application of the drug completely prevented the induction of LTP and its subsequent maintenance (Fig. 2G,H, S1, filled circles). Statistically significant potentiation was not observed either at any post‐tetanization time points when analyzed with its own baseline (Wilcox test, *P *>* *0.05) or with its control (*U*‐test, *P *>* *0.05). In short, NMDA receptor activity and protein synthesis are essential for the restoration of Aβ 1–42‐induced plasticity deficits by inhibition of G9a/GLP complex.

### G9a/GLP complex inhibition reinstates Aβ 1–42‐induced impairment of synaptic tagging/capture

**Figure 3:**
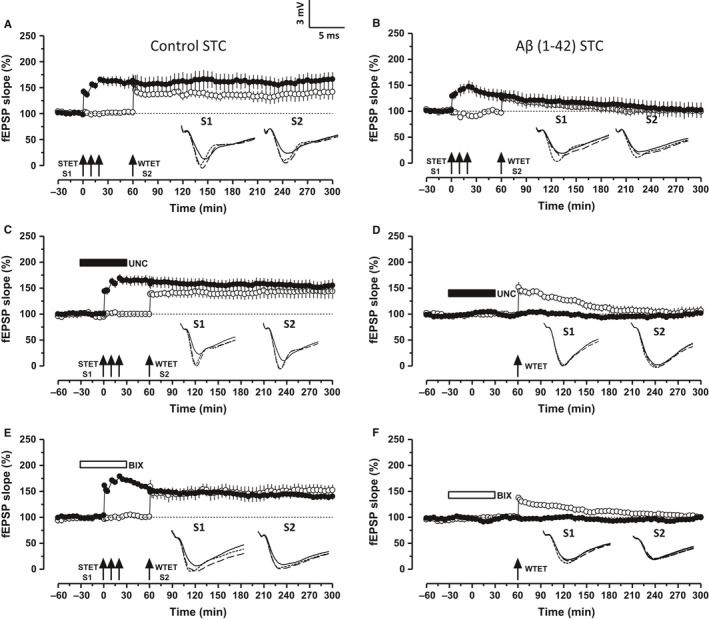
Inhibition of G9a/GLP complex ameliorates Aβ‐induced deficits in synaptic tagging/capture (STC). (A) STC was tested using ‘strong before weak’ (SBW) paradigm. Induction of early‐LTP in S2 (open circles) by WTET 60 min after the late‐LTP induction in S1 (filled circles) by STET resulted in late‐LTP in both the synaptic inputs, thereby expressing STC (*n* = 7). (B) The same SBW paradigm when used in the hippocampal slices pretreated with Aβ failed to express late‐LTP in both the inputs S1 and S2 (*n* = 6). (C, E) Inhibition of G9a/GLP complex activity by UNC 0638 (UNC, 150 nm) or BIX 01294 (BIX, 500 nm) restored the synaptic tagging and capture in Aβ‐treated slices. STET in the presence of either UNC or BIX resulted in late‐LTP in S1 (filled circles) and the early‐LTP in S2 (open circles) was transformed into late‐LTP (C, *n* = 8) (E, *n* = 8). (D,F) Application of WTET 30 min after the washout of UNC (D, 150 nm,* n* = 9) or BIX (F, 500 nm,* n* = 5) in S1 (filled circles) resulted in an early‐LTP. Control potentials in S2 (open circles) were stable during the recording period. Symbols and analog traces as in Fig. 1.

To test the effect of exogenous Aβ 1–42 oligomer on synaptic tagging/capture, we used the ‘strong before weak’ paradigm, in which STET was delivered to synaptic input S1 prior to WTET in S2 with an interval of 60 min (Frey & Morris, 1997, 1998). After a stable baseline of 30 min, STET was applied to S1 (Fig. 3B, filled circles) followed by WTET in S2 at 60th min (open circles). The slices treated with oAβ failed to express STC unlike the control, where both S1 and S2 (Fig. 3A) expressed a significant potentiation for 4 h (Wilcox test, *P = *0.018). In Fig. 3B, both the inputs S1 and S2 showed a statistically significant potentiation for 50 min (Wilcox test, *P = *0.046).

To investigate whether the inhibition of G9a/GLP complex activity could re‐establish STC, STET was applied to S1 (filled circles) in the presence of either of the substrate‐specific inhibitor of G9a/GLP complex, UNC (Fig. 3C) or BIX (Fig. 3E) followed by WTET in S2 (open circles) 60 min after the induction in S1. Both the synaptic inputs S1 and S2 showed a statistically significant potentiation that lasted until the end of the recording period (Fig. 3C,E, Wilcox test, *P = *0.0117), thereby expressing STC. To rule out the possibility that the G9a/GLP complex inhibitor itself reinforces the early‐LTP in synaptic input S2, control experiments were performed. After a stable baseline of 30 min in both S1 and S2, UNC (Fig. 3D) or BIX (Fig. 3F) was bath applied for 60 min. The drug was washed out for 30 min followed by WTET in S2. In both Fig. 3D,F, early‐LTP in S2 did not reinforce into late‐LTP. Synaptic input S2 showed a significant potentiation for 140 min (Wilcox test, *P = *0.021) in Fig. 3D and till 170 min (Wilcox test, *P = *0.046) in Fig. 3F.

**Figure 4:**
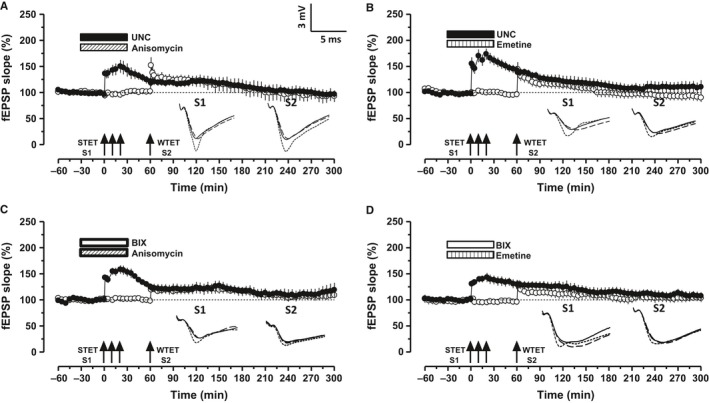
Protein synthesis inhibition during G9a/GLP inhibition prevents STC. (A–B) Co‐application of UNC with either of the protein synthesis inhibitors anisomycin (ANI 25 μm; A, *n* = 6) or emetine (EME 20 μm; B, *n* = 6) prevented the restoration of STC in Aβ‐treated slices. (C–D) Same experiment in A & B repeated with BIX (C, ANI,* n* = 7; D, EME,* n* = 6). Symbols and analog traces as in Fig. 1.

Synaptic tagging and capture involves the ‘capture’ of newly synthesized plasticity‐related products (PRPs) by the ‘tagged’ synapses. The re‐establishment of STC in oAβ‐treated hippocampal slices (Fig. 3C,E) could be due to the newly synthesized PRPs from the LTP restored by G9a/GLP complex inhibition in S1. These PRPs are sufficiently available to be captured by the synaptic tags set in S2 due to WTET, which then express late‐LTP. To confirm this hypothesis, STC experiments were replicated using anisomycin and emetine. Figure 4A–D represents STC experiments re‐established by G9a/GLP complex inhibition in the presence of either anisomycin (25 μm, Fig. 4A,C) or emetine (20 μm, Fig. 4B,D). In all cases, the hippocampal slices were pretreated with oAβ and protein synthesis inhibitors were co‐applied with either UNC or BIX. Protein synthesis inhibition along with either UNC or BIX in S1 not only prevented the restoration of late‐LTP but also the transformation of early‐LTP to late‐LTP in S2, eventually preventing expression of STC (Fig. 4A–D, filled circles and open circles). In Fig. 4A,B, statistically significant LTP was observed in S1 up to 145 min (Fig. 4A, filled circles, Wilcox test, *P = *0.04) and up to 105 min (Fig. 4B, filled circles, Wilcox test, *P = *0.046). S2 showed statistically significant LTP up to 85 min (Fig. 4A, open circles, Wilcox test, *P = *0.046) and up to 80 min (Fig. 4B, open circles, Wilcox test, *P = *0.046). Similarly, statistically significant LTP was observed in S1 up to 165 min (Fig. 4C, filled circles, Wilcox test, *P = *0.04) and up to 115 min (Fig. 4D, filled circles, Wilcox test, *P = *0.046). S2 showed statistically significant LTP up to 200 min (Fig. 4C, open circles, Wilcox test, *P = *0.017) and up to 105 min (Fig. 4D, open circles, Wilcox test *P = *0.046).

### Restoration of oAβ‐induced deficits in late‐LTP and STC by inhibition of G9a/GLP complex is established by BDNF

**Figure 5:**
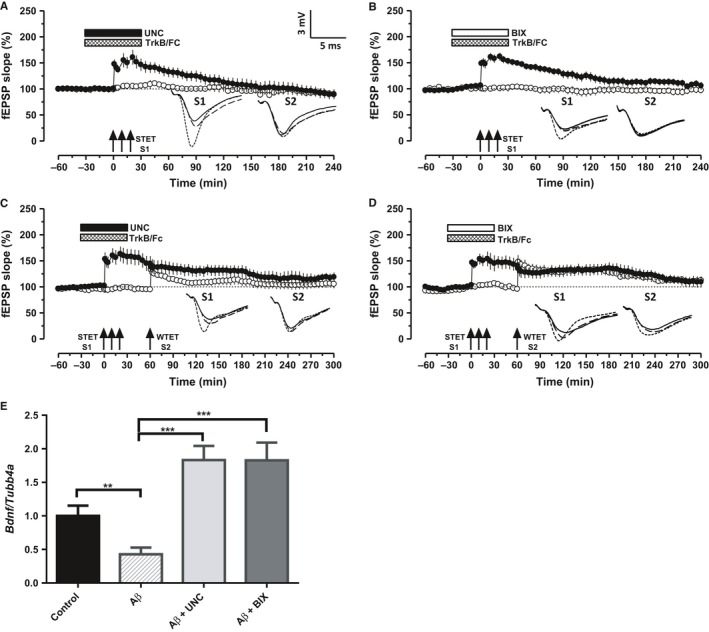
BDNF mediates the restoration of Aβ induced deficits in LTP and STC during the inhibition of G9a/GLP complex. A–B. Application of STET in the presence of BDNF chelator TrkB/Fc (1 μg mL^−1^) and either of the G9a/GLP inhibitors UNC (A, *n* = 7) or BIX (B, *n* = 7) failed to express a long‐lasting LTP (filled circles) in the Aβ‐treated slices. Control potentials (open circles) were stable throughout the recording. C–D. Inhibition of G9a/GLP complex failed to rescue Aβ‐induced deficits in STC when BDNF is chelated using TrkB/Fc. STET in S1 (filled circles) in the presence of UNC (C, *n* = 7) or BIX (D, *n* = 6) along with TrkB/Fc resulted in early‐LTP. Induction of early‐LTP in S2 60 min after the application of WTET in S1 also resulted in early‐LTP (open circles), hence no expression of STC. (E) *Bdnf* mRNA is downregulated in the Aβ‐treated hippocampal slices (*P *<* *0.01). Increased relative expression of *Bdnf* mRNA in CA1 region of Aβ‐treated hippocampal slices after UNC/BIX application for 1 h (one‐way ANOVA,* F* = 12.71; *P *<* *0.001). The values of the individual groups were calculated in relation to the control group. Each bar represents mean ± SEM (*n* = 6). Asterisk indicate significant difference (Bonferroni *post hoc* test, ***P <* *0.001). Symbols and analog traces as in Fig. 1.

To delineate the molecular underpinnings of the amelioration of oAβ‐induced LTP and STC deficits by the inhibition of G9a/GLP complex activity, a series of experiments were conducted using TrkB/Fc recombinant protein (1 μg mL^−1^) that prevents TrkB‐BDNF signaling. To validate the role of BDNF in rescuing the oAβ‐induced deficits in LTP and STC by G9a/GLP complex inhibition, TrkB/Fc was co‐applied with either UNC or BIX (Fig. 5A–D). Figure 5A,B used the experimental design as that of Fig. 2A to investigate the role of BDNF in LTP where STET was applied to induce late‐LTP 30 min after the bath application of drugs. Interestingly, restoration of late‐LTP by UNC or BIX was prevented resulting only in early‐LTP that lasted up to 85 min (Fig. 5A, filled circles, Wilcox test, *P = *0.043; *U*‐test, *P = *0.03) and up to 115–120 min (Fig. 5B, filled circles, Wilcox and *U*‐test, *P = *0.042).

To further probe the possible involvement of BDNF in re‐establishment of STC, the experimental design used in Fig. 4 was employed, but TrkB/Fc was co‐applied for 60 min either with UNC or BIX (Fig. 5C,D). Similar to Fig. 4A–D, potentiation in S1 decayed to baseline within 200 min (Fig. 5C, filled circles, Wilcox test, *P = *0.02) and up to 235 min (Fig. 5D, filled circles, Wilcox test, *P = *0.046). S2 exhibited early‐LTP as well, with a significant potentiation until 105 min (Fig. 5C, open circles, Wilcox test, *P = *0.042) and up to 210 min (Fig. 5D, open circles, Wilcox test, *P = *0.046). These results provide compelling evidence that BDNF‐TrkB signaling is imperative in restoring LTP and STC deficits by G9a/GLP inhibition.

To further confirm the above electrophysiology observations that G9a/GLP complex inhibition restores oAβ‐induced deficits in LTP and STC via BDNF, we quantified the gene expression of *Bdnf* normalized to the endogenous control *Tubb4a* (Tubulin 4a). qRT–PCR data revealed a significant increase in *Bdnf* expression following the induction of LTP in ‘Aβ + UNC’ and ‘Aβ + BIX’ groups when compared with either ‘control’ or ‘Aβ’ group (Fig. 5E; one‐way ANOVA, *F* = 12.71, *P *<* *0.001). A multiple comparison with Bonferroni *post hoc* test showed that the relative increase in *Bdnf* expression in ‘Aβ + UNC’ and ‘Aβ + BIX’ groups was statistically significant when compared to ‘control’ (*P *=* *0.03; *n* = 6) or ‘Aβ’ (*P *<* *0.001). We also observed a significant decrease in the *Bdnf* expression in ‘Aβ’ group as compared to the ‘control’ (*P *<* *0.01). Thus, G9a/GLP complex inhibition results in the upregulation of *Bdnf,* which enhances the plasticity and associativity in Aβ‐affected neural networks.

## Discussion

Dysregulation of epigenetic mechanisms is one of the major factors responsible for cognitive decline during aging and neurodegenerative diseases such as Alzheimer's disease (AD) (Cacabelos & Torrellas, 2015; Maloney & Lahiri, 2016). Substantial research has focused on rescuing the cognitive deficit during AD by regulating the histone acetylation in AD mouse models and *in vitro* studies (Cacabelos & Torrellas, 2015; Klein *et al*., 2015; Grinan‐Ferre *et al*., 2016; Krishna *et al*., 2016). Epigenetic regulation by G9a/GLP histone lysine–methyltransferase complex is emerging as a critical mechanism underlying the learning and memory processes (Schaefer *et al*., 2009; Maze *et al*., 2010; Gupta‐Agarwal *et al*., 2012). Our present findings confirm that inhibiting the catalytic activity of G9a/GLP complex is beneficial in restoring the late‐phase of LTP that is otherwise impaired by Aβ 1–42 oligomer (oAβ). The abrogation of late‐LTP by oAβ is consistent with previous studies (Ma *et al*., 2014; Lei *et al*., 2016). The synthesis of plasticity proteins, which is disrupted by Aβ 1–42 (Chen *et al*., 2002), is required for the maintenance phase of LTP (Frey *et al*., 1988; Pang & Lu, 2004). Restoring the protein synthesis capability of a neuronal population is therefore a plausible way to ameliorate the Aβ‐induced synaptic deficits. We demonstrate that rescue of oAβ‐induced LTP deficit by catalytic inhibition of G9a/GLP complex is indeed protein synthesis and NMDA receptor dependent, thus representing a physiological correlate of memory (Lynch, 2004).

Synaptic associativity is one of the unique features of healthy neural networks that encode memory traces on a long‐term basis (Redondo & Morris, 2011). The conversion of short‐term plasticity to long‐term plasticity widely relies on associative properties of a neural network. One characteristic feature of progressive dementia and AD is the deficit in associative plasticity or memory (Bastin *et al*., 2014; Jiang *et al*., 2015; Quenon *et al*., 2015). Our data provide the evidence that Aβ leads to deficits in synaptic tagging/capture (STC), which is considered as one of the major contributors of associative plasticity (Frey & Morris, 1997; Redondo & Morris, 2011). STC is characterized by two events: a) activity‐dependent ‘tagging of the synapses’, and b) ‘capture’ of the plasticity products by the synaptic tags (Redondo & Morris, 2011). The failure of expression of STC could be a result of disruption of either of these events or both. As it is widely accepted that the expression of any form of plasticity (weak or strong) is accompanied by the setting of a synaptic tag (Redondo & Morris, 2011), it can be hypothesized that the tagging of synapses during activity may be intact with exogenous administration of Aβ. Thus, the failure of expression of STC could be attributed to the disruption of active protein synthesis. To support this view, indeed we provide the evidence that the inhibition of G9a/GLP complex activity could re‐establish STC in a protein synthesis‐dependent manner.

Another intriguing observation from our study is the capability of Aβ oligomers to impair metaplastic nature of the synaptic populations. Metaplasticity is the capacity of a neuronal population to undergo or to prevent future plasticity, thus referring to the history of a synaptic population in plasticity and memory (Hulme *et al*., 2013). We have reported recently that metaplastic priming of G9a/GLP complex 30 min before or after the induction of early‐LTP can transform the transient plasticity to protein synthesis‐dependent long‐lasting form of plasticity (Sharma *et al*., 2016). Our data demonstrate that during Aβ 1–42 toxicity, priming by G9a/GLP complex inhibitors 30 min before the induction of early‐LTP was incapable of driving the synapses toward a metaplastic state. This line of data is consistent with the reports of aberrant metaplasticity during AD (Jang & Chung, 2016).

**Figure 6:**
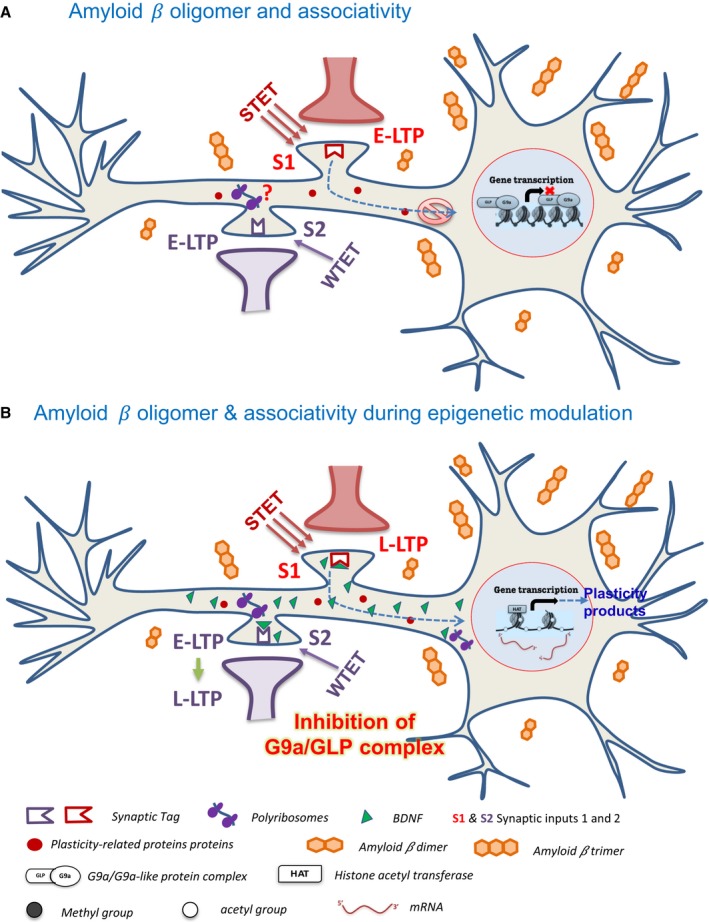
Schematic model representing the rescue of Amyloid β‐oligomer‐induced deficits in associative plasticity by inhibition of G9a/GLP complex. (A) The neuron when exposed to amyloid β oligomer, application of STET, marks the synapses (synaptic input 1, S1) with a synaptic ‘tag’ but fails to induce protein synthesis. The scarcity of plasticity proteins required for maintenance results in an early‐LTP. The subsequent induction of WTET in the neighboring synapses (S2) leads to an early‐LTP as well because of dearth of ‘plasticity proteins’ to be captured by the tagged synapses, resulting in the deficits in synaptic tagging/capture. Increased G9a/GLP activity is one of the contributing factors toward the malfunctioning of protein synthesis machinery during the presence of Aβ oligomer. G9a/GLP complex methylate the histones (H3K9me2) and nonhistone targets. H3K9me2 marks prevent the gene transcription by retaining the condensed form of chromatin that is inaccessible to the transcription machinery. This further interferes with the translation process of plasticity‐related proteins. (B) The pharmacological inhibition of G9a/GLP complex either by UNC0638 or BIX 01294 will prevent this complex from binding the histones and their subsequent methylation is intercepted. This will facilitate the binding of histone acetyl transferases (HATs), thereby resulting in the unwinding of DNA and rendering it accessible to the transcription machinery. This is followed by the increased expression of plasticity genes like *Bdnf*. The resulting upsurge in the translation process of plasticity‐related proteins like BDNF, which binds to TrkB receptor and activates downstream signaling pathways, paves the way to late‐LTP expression. The global distribution of BDNF and other plasticity proteins will enable the active set of synapses (S1 and S2) to ‘capture’ these proteins, resulting in a stable plasticity and late associativity. Additionally, the local synthesis of BDNF might be upregulated as well which further results in its profusion in the neuron. BDNF = brain‐derived neurotrophic factor; HAT = histone acetyltransferase; G9a/GLP = G9a/G9a‐like protein complex.

Histone 3 lysine 9 dimethylation (H3K9me2) by G9a/GLP facilitates DNA methylation (Shinkai & Tachibana, 2011) and hypermethylation of various genes implicated in synaptic plasticity and memory, including CREB‐regulated transcription co‐activator 1 (CRTC1) and brain‐derived neurotrophic factor (BDNF), is reported in the human hippocampus in AD (Nagata *et al*., 2015; Mendioroz *et al*., 2016). H3K9 methylation by G9a/GLP complex is a marker of gene silencing (Tachibana *et al*., 2005; Saksouk *et al*., 2015), and the repression of its activity has been shown to upregulate BDNF (Maze *et al*., 2010; Zhang *et al*., 2014). Our findings confirm that the inhibition of G9a/GLP complex releases the transcription silencing of *Bdnf* gene. The downregulation of *Bdnf* mRNA in the oAβ‐treated slices and the increased expression of *Bdnf* mRNA observed during catalytic inhibition of G9a/GLP complex indirectly suggest the heightened activity of G9a/GLP complex during Aβ 1–42 toxicity (Fig. 6). We further validate that re‐establishment of protein synthesis‐dependent plasticity and associativity is ensured by the availability of plasticity proteins, mainly BDNF. It strengthens our earlier findings from healthy neural system where the inhibition of G9a/GLP complex reinforced plasticity and associativity in physiological conditions via BDNF (Sharma *et al*., 2016).

Lower levels of BDNF correlate with poor cognitive status and higher amyloid‐beta protein (Beeri & Sonnen, 2016; Buchman *et al*., 2016). Soluble Aβ oligomers impair both vesicular and axonal BDNF trafficking resulting in disruption of BDNF signaling, which underlies the synaptic dysfunction displayed in AD (Poon *et al*., 2011; Seifert *et al*., 2016). BDNF plays a critical role in the formation and survival of neurons, maintenance of structural and functional plasticity and is a key player of plasticity and memory (Korte *et al*., 1995; Rex *et al*., 2007; Park & Poo, 2013). BDNF exerts protective effects against Aβ 1‐42‐induced neurotoxicity (Arancibia *et al*., 2008; Caccamo *et al*., 2010; Tejeda & Diaz‐Guerra, 2017). We provide evidence that BDNF‐TrkB signaling is crucial to the re‐establishment of Aβ‐induced deficits in plasticity and associativity by regulation of G9a/GLP complex. The binding of BDNF to its receptor TrkB is followed by the phosphorylation of TrkB that further triggers mainly three intracellular signaling cascades: (i) Phospholipase C‐γ (PLCγ)–Ca^2+^ pathway, (ii) phosphatidylinositol 3 kinase (PI3K)–Akt pathway, and (iii) the Ras–mitogen‐activated protein kinase (MAPK) pathway (Kaplan & Miller, 2000; Minichiello, 2009). It further results in either the increased postsynaptic density of AMPA receptors by PLCγ/PI3K–Akt pathway or the initiation of gene transcription of plasticity proteins by MAPK pathway (Pang & Lu, 2004; Minichiello, 2009). It is likely that one or multiple of pathways are acting synergistically to rescue or re‐establish the impaired plasticity and associativity in Aβ‐induced pathological situations (Fig. 6).

Together, our findings demonstrate that the regulation of G9a/GLP activity by inhibiting its activity rescues LTP and re‐establishes the expression of STC by increasing the BDNF signaling during Aβ pathology. Our results highlight the importance of epigenetic regulation of synaptic plasticity and associativity in the hippocampus by G9a/GLP complex in neuropathological situation and provide an evidence for the crucial role of BDNF in epigenetic regulation‐mediated rescue of synaptic deficits.

## Experimental procedures

### Electrophysiology

A total of 182 hippocampal slices prepared from 100 adult male Wistar rats (5–7 week old) were used for electrophysiological recordings. Animals were housed under 12‐h light/12‐h dark conditions with food and water available *ad libitum*. All experimental procedures using animals were performed in accordance with the protocols approved by the Institutional Animal Care and Use Committee (IACUC) of the National University of Singapore. Briefly, the rats were decapitated after anesthetization using CO_2_. The brains were quickly removed and cooled in 4 °C artificial cerebrospinal fluid (ACSF) that contained the following (in millimolars): 124 NaCl, 3.7 KCl, 1.0 MgSO_4_ .7H_2_O, 2.5 CaCl_2_, 1.2 KH_2_PO_4_, 24.6 NaHCO_3_ and 10 d‐glucose, equilibrated with 95% O_2_‐5% CO_2_ (carbogen; total consumption 16 L h^−1^). Transverse hippocampal slices (400 μm thick) were prepared from the right hippocampus using a manual tissue chopper. The slices were incubated at 32 °C in an interface chamber (Scientific System Design, Mississauga, ON, Canada) with an ACSF flow rate of 1 mL min^−1^.

In all the electrophysiological recordings, two‐pathway experiments were performed. Two monopolar lacquer‐coated stainless steel electrodes (5MΩ; AM Systems, Sequim, WA, USA) were positioned at an adequate distance within the stratum radiatum of the CA1 region for stimulating two independent synaptic inputs S1 and S2 of one neuronal population (Fig. 1A), thus evoking field EPSP (fEPSP) from Schaffer collateral/commissural‐CA1 synapses. Pathway specificity was tested using the method described in (Sajikumar & Korte, 2011). One electrode (5MΩ; AM Systems) was placed in the CA1 apical dendritic layer for recording the fEPSP. The signals were amplified by a differential amplifier (Model 1700; AM Systems), digitized using a CED 1401 analog‐to‐digital converter (Cambridge Electronic Design, Cambridge, UK), and monitored online.

After the pre‐incubation period, a synaptic input–output curve (afferent stimulation vs. fEPSP slope) was generated. Test stimulation intensity was adjusted to elicit fEPSP slope of 40% of the maximal slope response for both synaptic inputs S1 and S2. To induce late‐LTP, a ‘strong’ tetanization (STET) protocol consisting of three high frequency stimulations of 100 pulses at 100 Hz (single burst, stimulus duration of 0.2 ms per polarity), with an intertrain interval of 10 min, was used. To induce early‐LTP, a ‘weak’ tetanization (WTET) protocol consisting of a single stimulus train of 21 pulses at 100 Hz (stimulus duration of 0.2 ms per polarity) was used (Shetty *et al*., 2015). In all experiments, a stable baseline was recorded for at least 30 min using four 0.2‐Hz biphasic constant‐current pulses (0.1 ms per polarity) at each time point.

### Drugs

*In vitro* oligomer preparation of Aβ 1–42 peptide (AnaSpec, Fremont, CA, USA) and Aβ 42–1 peptide (Sigma‐Aldrich, Singapore, Singapore) was carried out 24 h before the start of experiment using the protocol mentioned in Stine *et al*. (2003). Briefly, Aβ (1–42 & 42–1) peptide films prepared in hexafluoroisopropanol (HFIP) were stored in −20 °C. The peptide films were dissolved in DMSO (dimethyl sulfoxide) followed by DMEM/F‐12 without phenol red and were then stored in 4 °C for 24 h to allow the oligomerization of the peptide. BIX 01294 (BIX; 270517, Enzo Life Sciences, Singapore) and UNC 0638 (UNC; U4885, Sigma‐Aldrich), the two selective and cell permeable inhibitors of G9a/GLP histone methyltransferase (Chang *et al*., 2009; Liu *et al*., 2010), were stored as 10 mm stocks in DMSO at −20 °C. The protein synthesis inhibitors, emetine dihydrochloride hydrate (Sigma‐Aldrich) and anisomycin (Tocris Biosciences, Bristol, UK) were stored as concentrated stock solutions of 20 mm in water and 25 mm in DMSO, respectively (Sajikumar *et al*., 2007). NMDA receptor antagonist AP5 (Tocris Biosciences, Bristol, UK) was stored as 50 mm stock solution in water. TrkB/Fc chimera human recombinant (TrkB/Fc; 688‐TK, R&D Systems, Minneapolis, MN, USA) was dissolved in sterile phosphate‐buffered saline (PBS) and stored at −20 °C (Chen *et al*., 2010). The stocks were stored for not more than a week. Just before application, the stocks were diluted to the final concentration in ACSF and bubbled with carbogen to be bath applied for specified durations. The final concentration used for Aβ (1–42 & 42–1), UNC, BIX, emetine, anisomycin, AP5 and TrkB/Fc was 200 nm, 150 nm, 500 nm, 20 μm, 25 μm, 50 μm and 1 μg mL^−1^, respectively. The light‐sensitive drugs were protected from light during storage and bath application. For the stocks prepared in DMSO, the final DMSO concentration was kept below 0.1%, a concentration that has been shown to not affect basal synaptic responses (Navakkode *et al*., 2004).

### Quantitative real‐time polymerase chain reaction (qRT–PCR)

For the qPCR analysis, four groups (each containing 6–8 slices) were collected from both right and left hippocampi of each of the six male Wistar rats. The groups were (i) ‘control’ – slices incubated in the interface chamber for 3 h; (ii) ‘Aβ’ – slices treated with 200 nm Aβ for 2 h followed by strong tetanization (STET); (iii) ‘Aβ + UNC’ – slices treated with Aβ were administered STET in the presence of G9a/GLP complex inhibitor UNC 0638 (UNC, 150 nm) where UNC was applied for 1 h; (iv) ‘Aβ + BIX’ – same as (iii) with the exception that another G9a/GLP complex inhibitor BIX 01294 (BIX, 500 nm) was bath applied. CA1 region was microdissected from all the hippocampal slices and flash‐frozen in liquid nitrogen and the samples were stored in −80 °C.

Total RNA was extracted from CA1 regions of the hippocampal slices using TRIzol^®^ RNA extraction method (Invitrogen, 15596018, Carlsbad, CA, USA) according to manufacturer's protocol and quantified using spectrophotometer (NanoDrop2000, Thermo Scientific, Singapore). The mRNA was reverse‐transcribed into complementary deoxyribonucleic acid (cDNA) using GoScript Reverse Transcription System (Promega, Cat No. A5000, Singapore). Briefly, 1 μg of RNA was subjected to preheating with 2 μL Oligo (dT) at 72 °C for 2 min. Reverse transcription was performed at 42 °C for 1 h. Further, StepOne Plus Real‐time PCR system (Applied Biosystems, Foster City, CA, USA) was used to carry out the qRT–PCR with Taqman universal PCR master mix (Cat. No. 4304437, Thermo Scientific) and TaqMan probes specific for *Bdnf* (Unigene: Rn11266; Assay ID: Rn02531967_s1, Thermo Scientific) and *Tubulin 4a* (*Tubb4a*, Ref Seq: NM_080882.1; Assay ID: Rn01758134_g1, Thermo Scientific). The qRT–PCR was performed in 96‐well plates with an initial denaturation at 95 °C for 10 min, followed by 40 amplification cycles each of 95 °C for 15 s, and 60 °C for 1 min. Each reaction was run in duplicate and analyzed following the standard ΔΔ*C*_t_ method using *Tubb4a* as a normalization control.

### Statistical analysis

All data are represented as Mean ± SEM. The average values of the slope function of the field EPSP (millivolts per milliseconds) expressed as percentages of average baseline values per time point were analyzed using the Wilcoxon signed rank test (Wilcox test) when comparing within one group and the Mann–Whitney *U*‐test (*U*‐test) when data were compared between groups. The nonparametric test was used because of the normality violation at small sample size. Statistical comparison for the qRT–PCR data was done using Student's *t*‐test and one‐way ANOVA with Bonferroni *post hoc* test. *P *<* *0.05 was considered as statistically significantly different (*P <* *0.05 **P <* *0.01 ***P <* *0.001). The statistical analyses were performed using the Prism software (GraphPad, San Diego, CA, USA).

## Funding

S.S. is supported by National Medical Research Council Collaborative Research Grant (NMRC/CBRG/0041/2013 and NMRC/CBRG/0099/2015) and NUS‐Strategic and Aspiration Research Funds. The funding agency had no role in design of experiments or its interpretation. M.S. is supported by President Graduate Fellowship, National University of Singapore.

## Conflict of interest

None declared.
